# Neuroregulatory mechanism of heat-sensitive moxibustion on the Dubi acupoint (ST 35) in patients with knee osteoarthritis: a resting-state functional magnetic resonance imaging study

**DOI:** 10.3389/fneur.2026.1699988

**Published:** 2026-02-09

**Authors:** Lu Tian, Tiansheng Lian, Hongwu Xie, Yanling Chen, Jing Zhang

**Affiliations:** 1 College of Acupuncture and Massage, Jiangxi University of Chinese Medicine, Nanchang, China; 2 Department of Rehabilitation, Wuping Country Hospital, Longyan, China; 3 Department of Rehabilitation, The Second Affiliated Hospital of Nanchang University, Nanchang, China; 4 Department of Acupuncture and Massage, The Affiliated Hospital of Jiangxi University of Chinese Medicine, Nanchang, China

**Keywords:** fractional amplitude of low-frequency fluctuation, heat-sensitive, knee osteoarthritis, moxibustion, neuroregulatory, resting-state functional magnetic resonance imaging

## Abstract

**Objective:**

To investigate the local brain functional changes after heat-sensitive moxibustion at the left ST35 (Dubi) acupoint in patients with knee osteoarthritis (KOA) based on resting-state functional magnetic resonance imaging (rs-fMRI), and to explore the possible neuroregulatory mechanisms of heat-sensitive moxibustion for pain relief using the fractional amplitude of low-frequency fluctuation (fALFF) analysis.

**Methods:**

A total of 30 KOA patients who were found to be insensitive to the heat of moxibustion in the non-heat-sensitive moxibustion (NHSM) group, and enrolled another 30 KOA patients with moxibustion sensation in the heat-sensitive moxibustion (HSM) group. Both groups received moxibustion at the left ST35 acupoint for 10 min (once daily for 10 consecutive days) at a distance of about 3 cm from the skin. Before the first treatment and after the tenth treatment, we assessed knee pain using visual analog scale (VAS) and performed rs-fMRI scans on the patients. The fALFF data of both groups were processed using the SPM 12 module of MATLAB software.

**Results:**

Compared with pre-moxibustion, the fALFF value of the HSM group in the frontal lobe, white matter, and left temporal lobe was significantly higher, while the occipital lobe and the right hemisphere was significantly lower. The region with the highest increase was the left temporal lobe, followed by white matter, and the region with the strongest decrease was the occipital lobe, followed by the frontal lobe and the right hemisphere. In the NHSM group, the fALFF value in the left occipital lobe, left medial frontal gyrus, left middle frontal gyrus, right superior frontal gyrus, right superior temporal gyrus, and right cerebellar posterior lobe was significantly lower, with the strongest decrease in the right cerebellar posterior lobe, followed by the right superior temporal gyrus. Compared with the NHSM group after treatment, the fALFF value of the HSM group in the external nucleus, white matter, right hemisphere, left cerebellum, and left hemisphere was significantly higher, and the frontal lobe, occipital lobe, and precentral gyrus was significantly lower. Additionally, a positive correlation was found between the fALFF changes of the left temporal lobe and the VAS score changes for each patient (pre- vs. post-treatment) in the HSM group (*r* = 0.764, *p* < 0.01), whereas a negative correlation was observed for the occipital lobe (*r* = −0.595, *p* < 0.01).

**Conclusion:**

This study reveals that the superior pain relief from heat-sensitive moxibustion is underpinned by a sensation-specific, bidirectional modulation of the brain’s pain-processing network. Unlike the generalized suppression observed in the NHSM group, the heat-sensitive state is characterized by a concerted increase in temporal lobe activity and decrease in occipital lobe activity, both changes being strongly predictive of individual clinical improvement. These results offer compelling neuroimaging evidence that the subjective heat-sensitive sensation reflects a more efficient and integrated brain state for analgesia.

**Clinical trial registration:**

https://www.chictr.org.cn/, ChiCTR2000033075.

## 1 Introduction

Knee osteoarthritis (KOA) is a progressive joint disease characterized primarily by degeneration of articular cartilage, destruction of hyaline cartilage, synovial inflammation, synovial hyperplasia, and subchondral bone sclerosis (1, 2). Clinically, patients predominantly present with knee swelling, pain, morning stiffness, and functional impairment that frequently necessitates assistive devices for mobility, severely compromising quality of life (3, 4). Notably, epidemiological studies reveal KOA accounts for 85% of the global osteoarthritis burden. As a degenerative joint disease, its prevalence increases markedly with age, affecting an estimated 16% of adults worldwide. It is particularly prevalent in the elderly and ranks among the leading causes of disability (5, 6). Current treatments for KOA primarily focus on alleviating symptoms rather than addressing the underlying pathology, such as oral nonsteroidal anti-inflammatory drugs (NSAIDs) in the early stage and joint replacement surgery or other surgical interventions for late stage patients (7). These interventions typically offer only temporary relief of symptoms and are frequently associated with high costs (e.g., in the United States alone, annual healthcare expenditures for KOA range from $5.7 billion to $15 billion) (8, 9). Therefore, the exploration of safe, effective, non-pharmacological treatment options with minimal side effects and cost-effectiveness has emerged as a significant focus in contemporary KOA research.

As a non-invasive procedure, moxibustion applies thermal stimulation via ignited moxa or mugwort (*Artemisia vulgaris*) to acupoints directly or indirectly in order to attenuate pain or accelerate recovery in many diseases, particularly in chronic diseases (10, 11). Although moxibustion is less integrated into mainstream Western neurological and pain management practice—often due to differences in medical paradigms, evidence thresholds, and practical application—it is a well-established therapy in East Asian regions such as China, Japan, and Korea, valued for its symptomatic efficacy, convenience, and cost-effectiveness in managing conditions like KOA (12, 13). The findings of several meta-analyses and animal experiments have indicated that moxibustion has its unique advantages for KOA patients with its analgesic and anti-inflammatory effects (14), thereby effectively alleviating articular cartilage damage and improving joint function in early-stage osteoarthropathy (15). Importantly, clinical evidence further indicates that early intervention with moxibustion significantly, by alleviating pain, mitigating inflammation, and potentially slowing cartilage degeneration, can effectively disrupt the pathological progression of KOA, thereby significantly reducing the incidence of late-stage joint deformities (16). Heat-sensitive moxibustion, an advanced technique grounded in classical moxibustion principles, incorporates the theory of acupoint sensitization (a state of neuroplasticity involving altered nociceptive signaling). By targeting sensitized acupoints exhibiting heightened thermal responsiveness, this approach amplifies therapeutic efficacy (17). Multiple studies have demonstrated its superior clinical outcomes in KOA management, showing greater improvements in pain relief, physical function, and joint mobility compared to conventional moxibustion or standard care (18–20). However, a pivotal neurophysiological question remains regarding whether heat-sensitive moxibustion induced analgesia in KOA patients engages distinct central neuroregulatory mechanisms between thermo-sensitive and non-thermo-sensitive acupoint states. Elucidating this distinction could refine mechanism-driven application of acupoint sensitization theory.

Modern neuroimaging studies have revealed that the brain exhibits organized and coherent functional networks during resting-state, collectively termed the default mode network (DMN) (21). Building upon this framework, the pain-default network emerges as a specialized neural architecture that integrates nociceptive perception, pain modulation, and chronic pain adaptation, forming a dynamic system for pain-related information processing and integration (22). Rs-fMRI, characterized by non-invasiveness, high spatiotemporal resolution, and operational stability (23–28), offers a neuroimaging window to directly capture baseline brain functional architecture and its dynamic reorganization. This modality has been instrumental in objectively investigating the neurophysiological underpinnings of heat-sensitive moxibustion (29). In this study, we employed the fractional amplitude of low-frequency fluctuation (fALFF) analysis to decode blood oxygen level-dependent (BOLD) signal dynamics in KOA patients undergoing heat-sensitive moxibustion at the ST35 (Dubi) acupoint. By comparing region-specific brain functional plasticity between acupoint sensitization states (thermo-sensitive) and non-sensitized states, we aim to delineate differential neuroregulatory mechanisms engaged during analgesia. These findings provide preliminary neuroimaging evidence that may help elucidate the central mechanisms of acupoint sensitization. They suggest the potential for sensation-specific neuromodulation and could inform the future development of optimized non-pharmacological strategies for KOA pain.

## 2 Materials and methods

### 2.1 Participants

**Figure 1:**
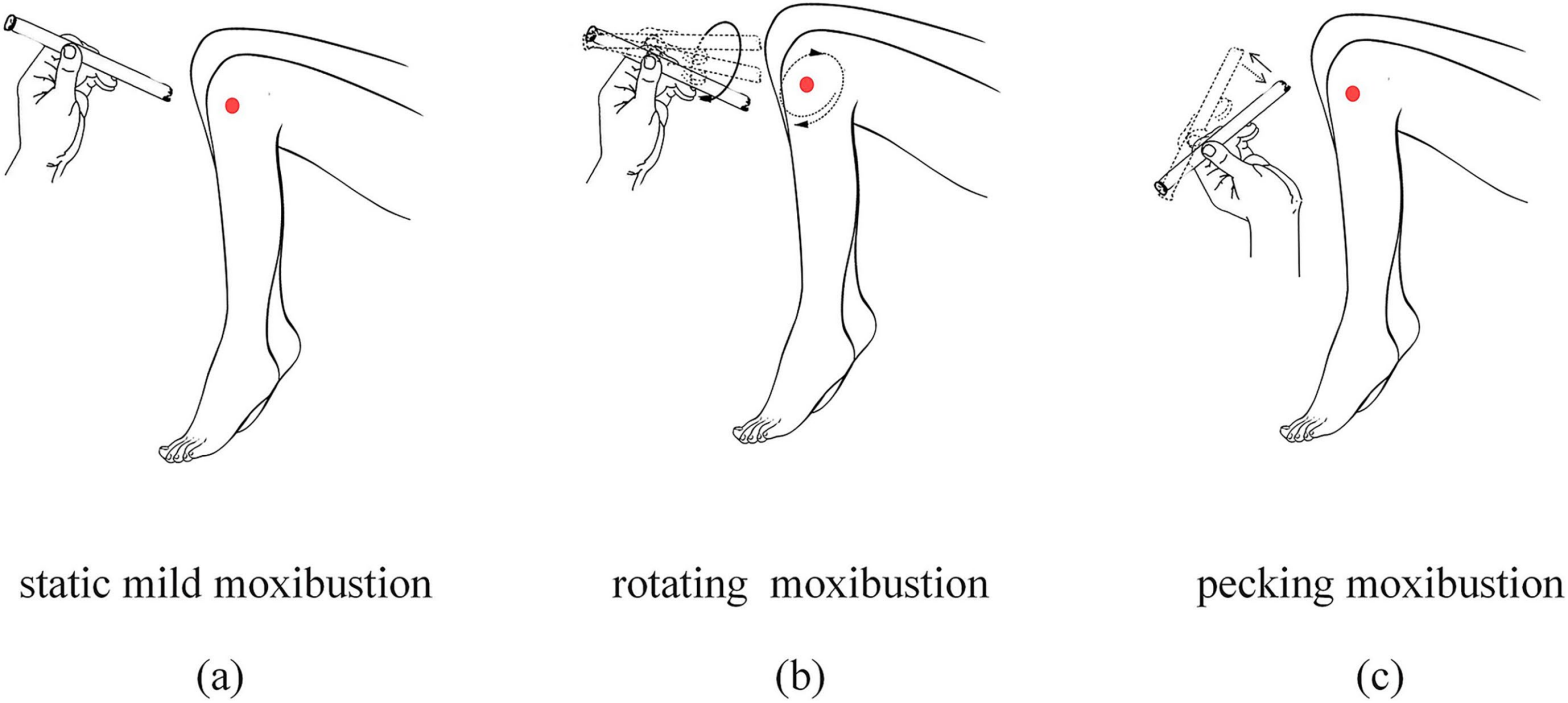
Schematic diagram of moxibustion techniques. **(a)** Static mild moxibustion: Ignite one end of the moxa stick and position it 2–3 cm above the target acupoint. Maintain stationary fumigation until the patient experiences localized warmth without burning sensation, holding the stick steadily over the acupoint; **(b)** Rotating moxibustion: Hold the ignited moxa stick 2–3 cm above the acupoint. Uniformly rotate the stick in slow, continuous circular motions to fumigate the acupoint area; **(c)** Pecking moxibustion: Position the ignited end of the moxa stick 2–3 cm above the acupoint. Rhythmically move the stick upward and downward in a vertical plane, mimicking a sparrow’s pecking motion to fumigate the acupoint.

**Figure 2:**
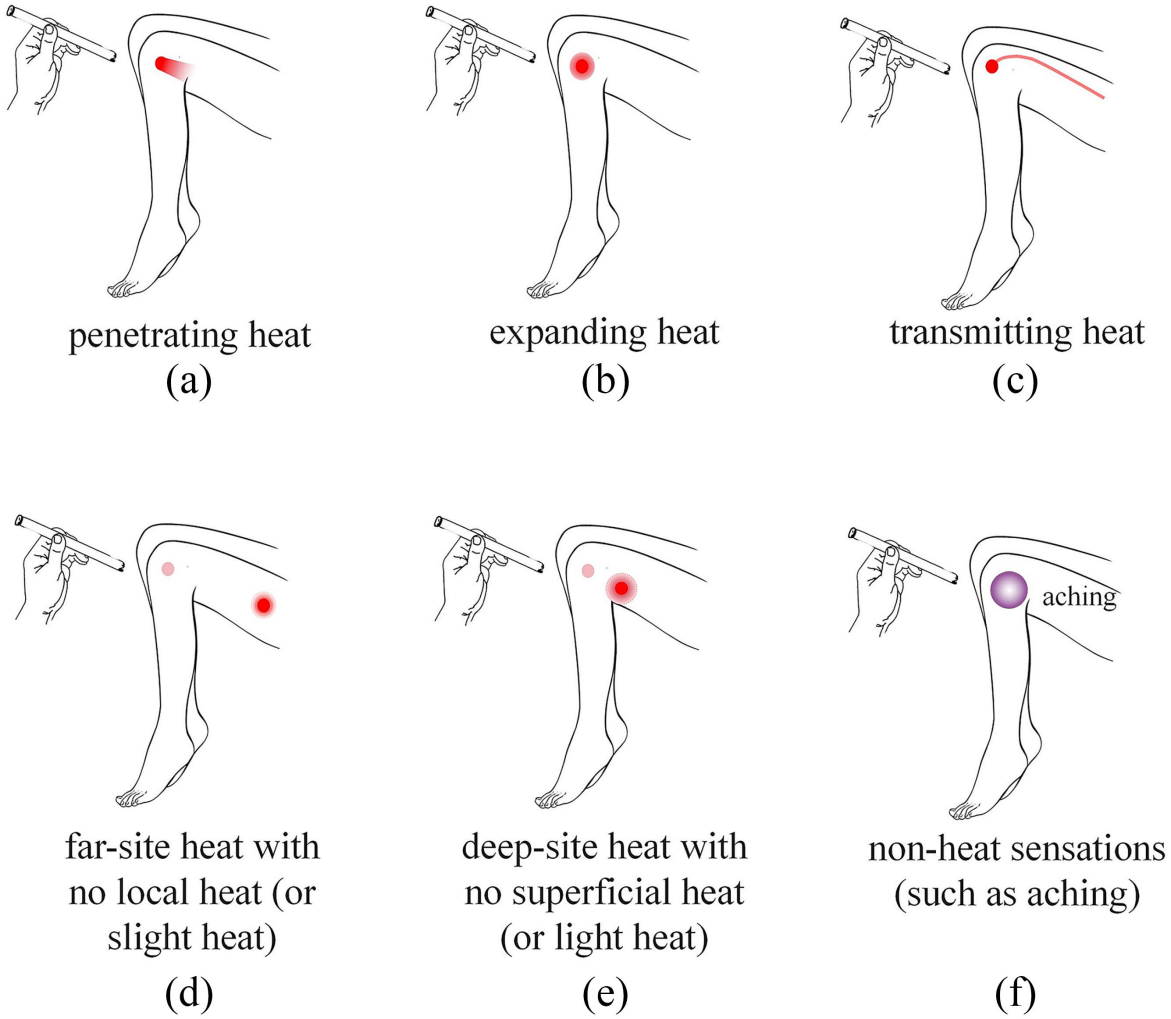
Schematic diagram of heat-sensitive moxibustion feelings. **(a)** Penetrating heat: the heat sensation conducting from the moxa local skin surface into deep tissue; **(b)** expanding heat: the heat sensation spreading to the surrounding little by little around the moxa point; **(c)** transmitting heat: the heat sensation transferring along some pathway or direction; **(d)** far-site heat with no local heat (or slight heat): the heat sensation perceived distally or at a distant site without noticeable heat at the moxa point or the local skin surface; **(e)** deep-site heat with no superficial heat (or light heat): the heat sensation distinctly perceived in deep tissues, muscles, or even internal organs, while the skin surface at or around the moxa point feels only mild warmth or no noticeable heat at all; **(f)** non-heat sensations: instead of thermal sensations, some patients perceive aching, heaviness, pain, numbness, pressure, or cold in local or distant locations of stimulation.

A total of 72 KOA patients were consecutively recruited from the Acupuncture Department (outpatient and inpatient units) of Jiangxi University of Chinese Medicine Affiliated Hospital between October 2023 and April 2024. All participants underwent standardized thermal responsiveness screening (19): initial 1-min static mild moxibustion at target acupoints followed by rotating and pecking techniques to provoke sensitization responses (categories a to c in Figure 1). Subjects demonstrating at least one predefined moxibustion feeling (categories a to f in Figure 2) during acupoint stimulation were allocated to the heat-sensitive moxibustion (HSM) group, whereas those failing to exhibit any of these moxibustion feelings were assigned to the non-heat-sensitive moxibustion (NHSM) group. This study identified 30 subjects exhibiting non heat-sensitive feeling responses, who were subsequently assigned to the NHSM group. To ensure methodological rigor, an equivalent number of participants (*n* = 30) demonstrating heat-sensitive responses were allocated to the HSM group through matched enrollment based on predefined experimental criteria.

### 2.2 Diagnostic criteria

All enrolled participants were diagnosed by two certified rheumatologists through a standardized pathway adhering to the American College of Rheumatology (ACR) 2012 criteria for KOA, integrating clinical assessment (history/symptoms/signs) and paraclinical evidence (imaging/ancillary investigations) (30–32).

Diagnostic criteria included:(1) recurrent knee pain within the preceding month; (2) radiographic confirmation of osteophyte formation combined with positive patellar ballottement test; (3) age ≥40 years; (4) morning stiffness duration ≤30 min; (5) audible crepitus with restricted flexion or rotational movement. Definitive diagnosis required fulfillment of either: (i) criteria (1) + (4) + (5); (ii) criteria (1) + (2) + (5); or (iii) criteria (1) + (2) + (3) + (4).

### 2.3 Inclusion criteria

(1) Meeting the above KOA diagnostic criteria with bilateral involvement; (2) Aged between 40 and 70 years old; (3) Right-handed dominance; (4) Absence of neurological diseases or psychiatric disorders (5) Persistent knee pain >3 months; (6) Willingness to provide written informed consent.

### 2.4 Exclusion criteria

(1) Suffering from chronic pain conditions such as myofascial pain syndrome, cervical spondylosis, intervertebral disc protrusion, postherpetic neuralgia, periarthritis of the shoulder; (2) Having pathological changes in the brain, such as cerebral infarction, brain infection, brain tumor; (3) Having comorbidities such as hypertension, diabetes, or other chronic diseases; (4) Having cognitive impairment; (5) Having skin diseases or secondary infections in the joint area; (6) Having a tendency to bleed, such as thrombocytopenia, allergic purpura; (7) Being allergic to moxibustion; (8) Having claustrophobia.

### 2.5 Withdrawal/elimination criteria

(1) During the trial, trauma occurs, or symptoms such as increased knee pain and stiffness due to worsening of the condition; (2) Burns caused by improper operation or other adverse reactions occur; (3) During the trial, the subject receives other treatments without permission or has poor compliance; (4) The subject voluntarily requests to terminate the trial.

### 2.6 Sample size

The calculation was based on efficacy rates reported in prior studies (85% for heat-sensitive moxibustion vs. 45% for the control intervention). We determined a requirement of 23 participants per group, with *α* = 0.05 and *β* = 0.10 (power = 85%), calculated using PASS 11.0. Considering an estimated 15% dropout rate, the target recruitment was set at 30 participants per group (HSM and NHSM).

### 2.7 Blinding

All clinical outcomes and all neuroimaging (fMRI) data preprocessing and statistical analyses were performed by researchers completely blinded to group allocation.

### 2.8 Acupoint selection and moxibustion

**Figure 3:**
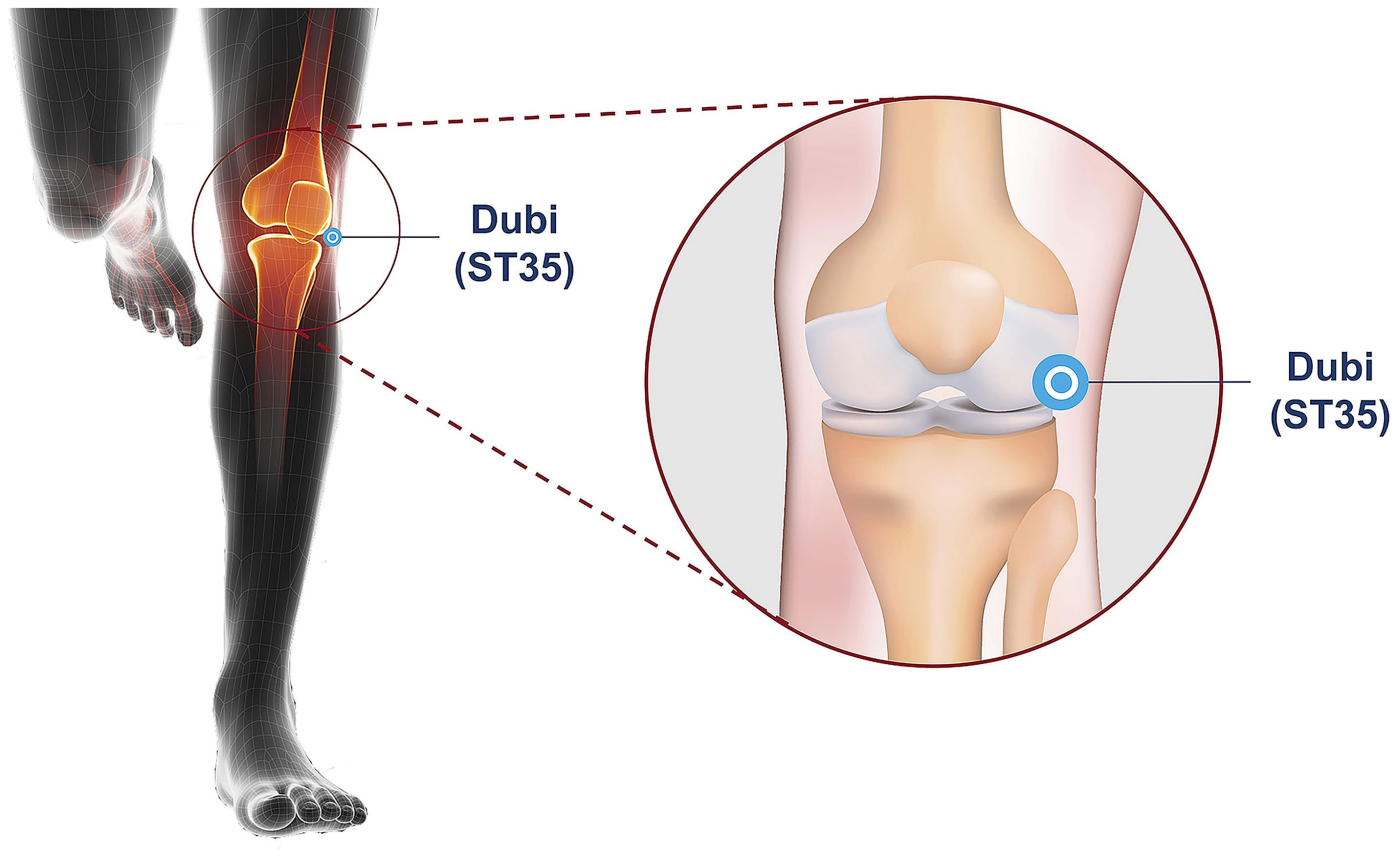
Location of acupoint ST35 (Dubi).

Moxibustion was applied exclusively at the ST35 (Dubi) acupoint, anatomically localized to the lateral depression of the patellar ligament in the anterior knee region. Precise anatomical coordinates were determined according to the National Standard of the People’s Republic of China (GB/T 12,346–2006) (33), with standardized positioning demonstrated in Figure 3.

All patients received standardized conventional therapy throughout the trial period to mitigate outcome bias from variable rehabilitation protocols across outpatient and inpatient settings. Both groups underwent identical rehabilitation interventions including health education, weight management, 5 sets/day of hamstring curls and wall squats, and prescribed assistive devices to improve balance/stability during ambulation; critically, no pharmacological interventions were permitted during the study.

**Figure 4:**
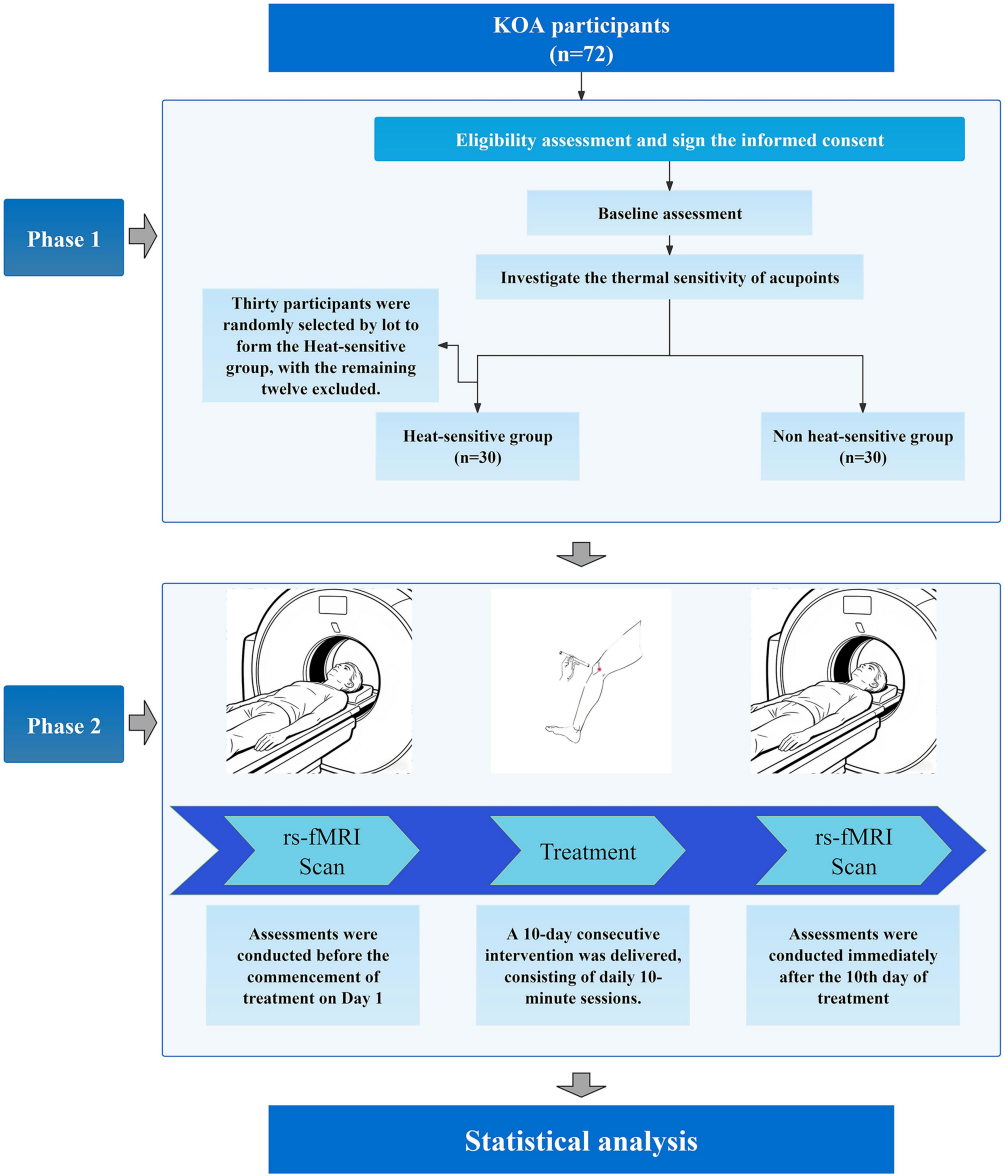
Flowchart.

The moxibustion strategies were developed by senior researchers of moxibustion and China acupuncture experts, based on the textbook and our previous study conclusions (19, 34, 35). Physicians trained and experienced (at least 5 years) in acupuncture delivered the interventions. Moxibustion was performed under controlled conditions (26–30 °C, quiet environment). Participants assumed a supine position with full exposure of the target area. Certified acupuncturists applied smokeless moxa sticks (Ø 22 mm × 120 mm; Batch No. 20211211; Hubei AiZhongAi Herb Technology Co., Ltd.) at ST35 (Dubi) on the left knee. Practitioners applied initial thermal stimulation 1–3 cm above ST35 acupoint until heat sensation was reported, followed by static moxibustion maintained at 3 cm height. The skin temperature at the stimulation site was monitored in real-time using an infrared thermography camera and maintained within a safe and effective range of 42–48 °C by fine-adjusting the moxibustion height. In the HSM group, 10-min static moxibustion commenced upon confirmation of moxibustion feelings; In the NHSM, Immediate 10-min static moxibustion at 3 cm. As shown in Figure 4, both groups received once daily treatment over 10 consecutive days. For breakthrough pain, rescue analgesia with celecoxib capsules (200 mg/day; China FDA Approval No. H20203325; Qingdao Baiyang Pharmaceutical Co., Ltd.) was administered, with detailed recording of medication usage and adverse reactions.

### 2.9 Pain intensity rating

The visual analog scale (VAS) was used to evaluate pain intensity. KOA patients were asked to mark a 10 cm line to represent the intensity of their pain. Scores could range from 0 to 10, 1 cm for 1 point; 0 points stood for no pain and 10 points for the most intense pain. For patients with KOA, a VAS reduction of ≥1.5 points is generally considered the minimal clinically important difference (MCID), indicating a change perceived as meaningful.

### 2.10 fMRl data acquisition

On the first day before moxibustion and the tenth day after moxibustion, patients in both groups were scanned using a 3.0-Tesla Discovery MR750 Scanner (GE Healthcare, United States). The scanning lasted for 8 min. Before scanning, it was ensured that the subjects were not wearing metal accessories and did not have any implants in their bodies. The subjects’ heads were fixed with sponges or soft pillows to reduce head movement, and earplugs were worn to reduce noise, ensuring a comfortable and quiet environment for the subjects. During fMRI scanning, subjects lay supine with their eyes closed, rested and remained conscious, avoiding emotional fluctuations and movements of the head and body. If a subject fell asleep or became confused after scanning, their fMRI data were excluded.

(1) Structure image scanning used a T1 weighted image sequence (3D-T1BRAVO): repetition time/echo time (TR/TE) = 11 ms/3.2 ms, slice thickness = 1.1 mm, number of slices = 225, slice spacing = 0 mm, flip angle (FA) = 45°, field of view (FOV) = 250 mm × 250 mm, resolution = 1 mm. (2) T2-FGRE sequence parameters: TR = 3,351 ms, FOV = 24 cm × 24 cm, imaging time = 2 min 5 s, TE = 102.9 ms. (3) A-BOLD sequence parameters: TR = 2000 ms, number of slices = 45, TE = 30 ms, slice thickness = 2 mm, matrix = 70 × 70 × 40, FOV = 250 mm × 250 mm, slice spacing = 0 mm, imaging time = 8 min, number of excitations = 1, flip angle = 45°, acquiring 180 time points.

### 2.11 Data pre-processing and data analysis

Data preprocessing was performed using DPARSF V2.2 and SPM 12 software.^1^ The preprocessing steps were as follows (36): (1) Data format conversion: DICOM (Digital Imaging and Communications in Medicine) to NIFTI (Neuroimaging Informatics Technology Initiative); (2) Removal of the first 10 time points; (3) Head motion correction: Subjects with head motion exceeding 2.5°, translation exceeding 2.5 mm, or mean FD_Jenkinson > 0.2 were excluded. Manual adjustments were made to achieve standardization; (4) Anatomical image segmentation; (5) Image registration; (6) Spatial normalization; (7) Removal of linear drift; (8) Spatial smoothing: A Gaussian smoothing kernel with a full width at half-maximum (FWHM) of 6 mm was applied; (9) Filtering: Bandpass filtering at 0.01–0.10 Hz (37).

Calculation of fALFF: The time series of each voxel was converted to the frequency domain using a fast Fourier transform algorithm to obtain the power spectrum. The average square root of the power spectrum within the 0.01–0.10 Hz range was then calculated as the amplitude of low-frequency fluctuation (ALFF) value, and the ALFF values of each participant were converted to z-scores. Furthermore, the ratio of ALFF to the root mean square of the full-frequency power spectrum was computed to derive the fALFF value. The value of fALFF, calculated as the ratio of low-frequency amplitude to whole-brain amplitude, reflects the excitability and spontaneous neuronal activity of brain regions. Higher fALFF values indicate greater excitability and stronger spontaneous neuronal activity, while lower values suggest functional suppression (38, 39).

Multiple comparison correction was performed using AlphaSim with the following parameters: (1) Edge connection with radius *r* = 5 mm; (2) Smoothing kernel size: 6 mm × 6 mm × 6 mm; (3) Single-sample *t*-test union as a mask, corrected *p* < 0.01. The DPABI Viewer V4.3 software was used to visualize the specific anatomical locations of brain regions corresponding to the MNI (Montreal Neurological Institute) coordinate system in the three-dimensional human brain coordinate system.

### 2.12 Statistical analysis

Statistical analyses were performed using SPSS 22.0. Continuous data, which followed a normal distribution, were expressed as mean ± standard deviation ( $\bar{x}\;$ ± *s*). For data with homogeneous variance, independent-sample *t*-tests or paired-sample *t*-tests were used; For data with heterogeneous variance, *t*-tests were applied. Categorical data were expressed as frequencies and analyzed using the Chi-square test. Paired sample *t*-tests were used to compare pre- and post-treatment data within groups. A 2-way repeated-measures analysis of variance was used to analyze the group × time interaction. SPM12 software was used to perform dual sample t tests, paired t tests on fALFF values to obtain significant differences. Multiple comparison corrections were performed using Rest Slice Viewer, and brain regions with a cluster volume threshold of ≥60 voxels and a single-voxel threshold of *p* < 0.05 (corrected) were defined as regions with statistically significant differences. Brain regions with significant fALFF differences between the HSM and NHSM groups were identified as regions of interest (ROIs). To examine the relationship between alterations in brain activity and clinical efficacy, a correlation analysis was performed between the fALFF values changes of these ROIs and the changes in VAS scores for each patient from pre- to post-treatment, using the Pearson correlation analysis, *p* < 0.05 was considered statistically significant.

## 3 Results

### 3.1 Demographic and clinical data

The HSM group comprised 17 males and 13 females, with an average age of 60.54 ± 10.83 years, and disease duration of 3.57 ± 1.69 years. The NHSM group included 21 males and 9 females, with an average age of 61.69 ± 10.97 years, and disease duration of 3.81 ± 1.59 years. Between-group differences in demographic and clinical characteristics (gender, age, disease duration) were compared using parametric analyses: continuous variables underwent one-way ANOVA with post-hoc *t*-tests, while categorical data were analyzed via chi-square tests. No statistically significant intergroup disparities were observed (*p* > 0.05), confirming baseline comparability between cohorts (Table 1).

**Table 1:** Demographic and clinical traits of all participants.

Group	Gender (people)		Age (years)		Course of KOA (years)	
	Male	Female	Range	Mean, SD	Range	Mean, SD
HSM	17	13	46~72	60.54 ± 10.83	1.7~5.5	3.57 ± 1.69
NHSM	21	9	45~73	61.69 ± 10.97	1.6~5.5	3.81 ± 1.59

HSM, the heat-sensitive moxibustion group; NHSM, the non-heat-sensitive moxibustion group; KOA, knee osteoarthritis; SD, standard deviation. *N* = 30; To identify demographic difference between groups ANOVA and *t*-tests were performed for continuous data. Chi-square tests were performed for categorical data. There are no statistical difference no statistically significant differences in terms of gender, age, and disease duration, *P* > 0.05.

### 3.2 VAS scores data

As shown in Table 2, a 2-way repeated-measures ANOVA performed on the VAS scores revealed a statistically significant Group × Time interaction effect (*F* = 11.43, *p* = 0.001). To decompose this significant interaction, simple effects analyses were conducted. The results indicated a significant reduction in pain after treatment within both the HSM group (*p* < 0.001) and the NHSM group (*p* = 0.031). Furthermore, while no significant difference in pain scores was observed between the two groups at baseline (*p* = 0.295), the HSM group demonstrated significantly lower pain scores than the NHSM group at the post-treatment time point (*p* < 0.001).

**Table 2:** Descriptive statistics and repeated-measures ANOVA for VAS scores.

Group	n	Pre-moxibustion	Post-moxibustion	Main and interaction effects	*F-*value	*P*-value
HSM	30	7.34 ± 1.80	3.64 ± 0.58			
NHSM	30	6.28 ± 1.82	5.62 ± 0.74			
Effect				Group	4.12	0.048
				Time	85.71	<0.001
				Group × Time	11.43	<0.001

HSM, the heat-sensitive moxibustion group; NHSM, the non-heat-sensitive moxibustion group; n, number of patients; VAS, visual analog scale. Repeated-measures ANOVA were performed for pre- and post-moxibustion comparisons within the NHSM group and the HSM group.

### 3.3 Result of the fALFF analyses

#### 3.3.1 The change of fALFF value in the HSM group

**Figure 5:**
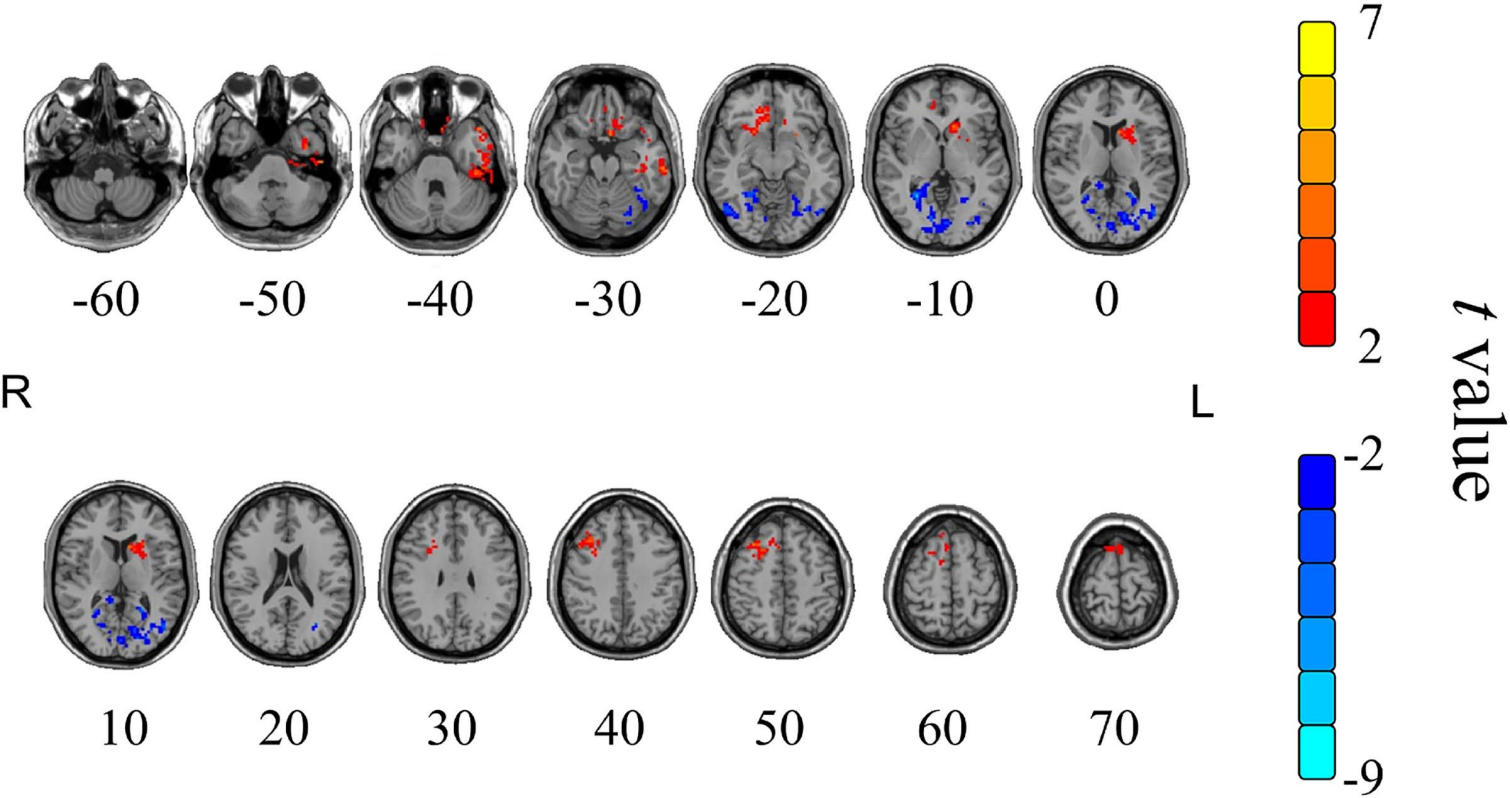
Cerebral regions with significant changes of fALFF value in the HSM group after moxibustion. Color scale denotes the *t*-value, yellow and red represent the brain area with increased fALFF, while blue represents the brain area with decreased fALFF; R represents right in the brain, L represents left in the brain; the paired *t*-tests, single voxel threshold *p* < 0.05, cluster volume threshold for the 85 continuum element K value greater than or equal to 85.

In the HSM group, the value of fALFF changes between the pre-moxibustion and post-moxibustion are shown in Table 3 and Figure 5. Compared to pre-moxibustion statue, brain regions with significantly increased fALFF were the frontal lobe, white matter, and left temporal lobe. Regions with significantly decreased fALFF were the occipital lobe and the right hemisphere. The region with the highest increase in fALFF was the left temporal lobe, followed by white matter. The region with the strongest decrease was the occipital lobe, followed by the frontal lobe and the right hemisphere.

**Table 3:** Cerebral regions with significant changes of fALFF value in the HSM group after moxibustion.

Cerebral regions	Voxels	Peak MNI coordinates			*t*-value (peak)	*P*-value
		X	Y	Z		
Frontal lobe	116	39	24	42	6.31	<0.001
White substance region	115	6	39	−6	6.30	<0.001
Left temporal lobe	159	−48	18	27	8.64	<0.001
Occipital lobe	494	−39	−51	−24	−8.81	<0.001
Right cerebral	109	45	−27	−9	−4.50	0.003

MNI coordinates, Montreal Neurological Institute coordinates of the peak voxel; *t-*values: statistical value of the peak voxel, *P* < 0.05; Alphasim correction.

#### 3.3.2 The change of fALFF value in the NHSM group

**Figure 6:**
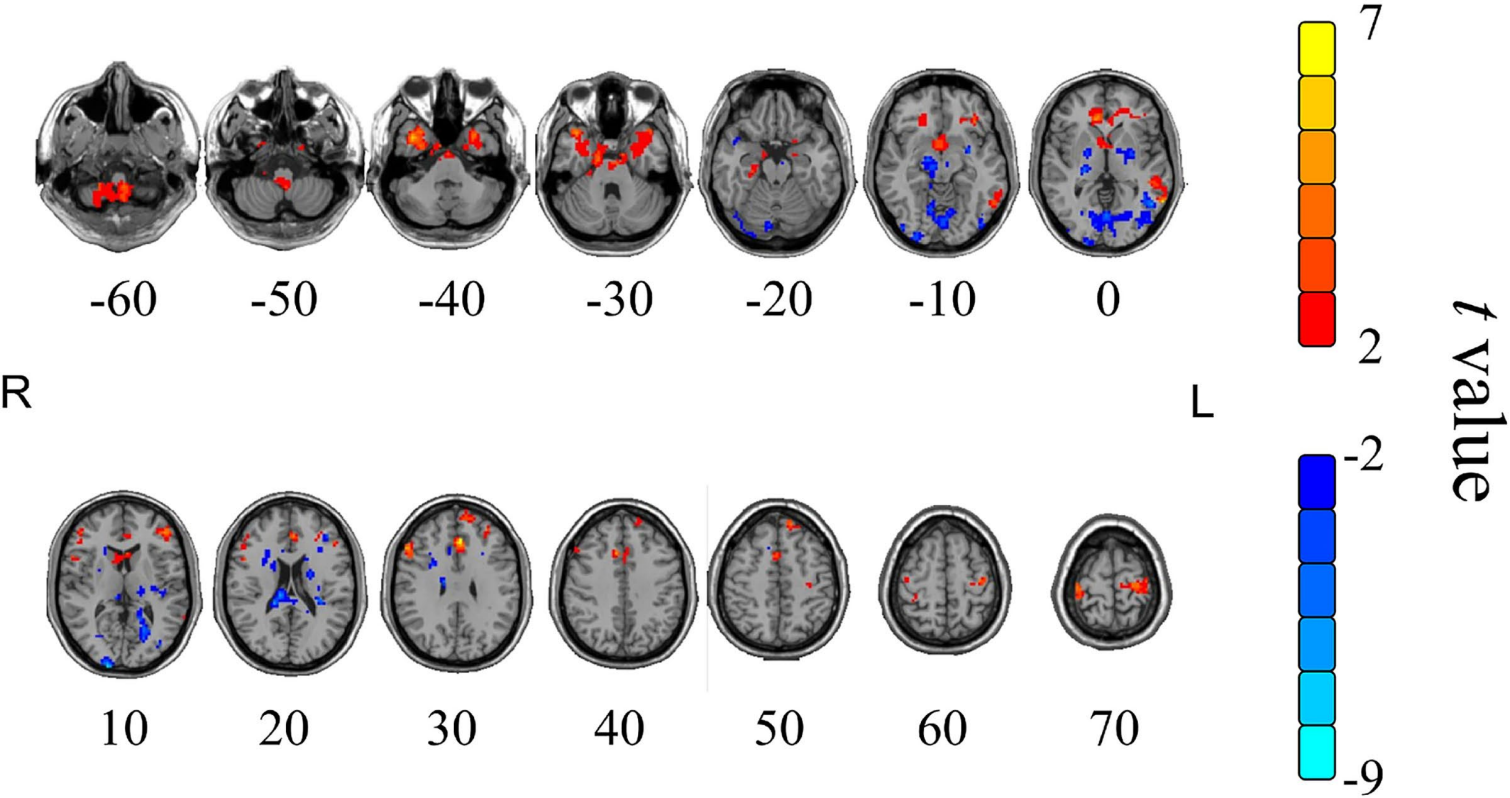
Cerebral regions with significant changes of fALFF value in the NHSM group after moxibustion. Color scale denotes the *t*-value, yellow and red represent the brain area with increased fALFF, while blue represents the brain area with decreased fALFF; R represents right in the brain, L represents left in the brain; he paired *t*-tests, single voxel threshold *p* < 0.05, cluster volume threshold for the 85 continuum element *K* value greater than or equal to 85.

In the NHSM group, the value of fALFF changes between the pre-moxibustion and post-moxibustion are shown in Table 4 and Figure 6. Compared to the pre-moxibustion statue, brain regions with significantly decreased fALFF were the left occipital lobe, left medial frontal gyrus, left middle frontal gyrus, right superior frontal gyrus, right superior temporal gyrus, and right posterior cerebellar lobe. The region with the strongest decrease in fALFF was the right posterior cerebellar lobe, followed by the right superior temporal gyrus.

**Table 4:** Cerebral regions with significant changes of fALFF value in the NHSM group after moxibustion.

Cerebral regions	Voxels	Peak MNI coordinates			*t*-value (peak)	*P*-value
		X	Y	Z		
Left occipital lobe	149	−30	−57	3	−6.62	<0.001
Left medial frontal gyrus	97	0	−27	69	−4.37	0.002
Left middle frontal gyrus	98	−27	54	−6	−4.73	<0.001
Right superior frontal gyrus	109	33	57	−3	−4.80	<0.001
Right superior temporal gyrus	83	51	−18	3	−6.53	<0.001
Right posterior lobe of cerebellum decline	85	42	−63	−24	−8.64	<0.001

MNI coordinates, Montreal Neurological Institute coordinates of the peak voxel; *t*-values: statistical value of the peak voxel, *P* < 0.05; Alphasim correction.

#### 3.3.3 Comparison between the HSM and NHSM groups

**Figure 7:**
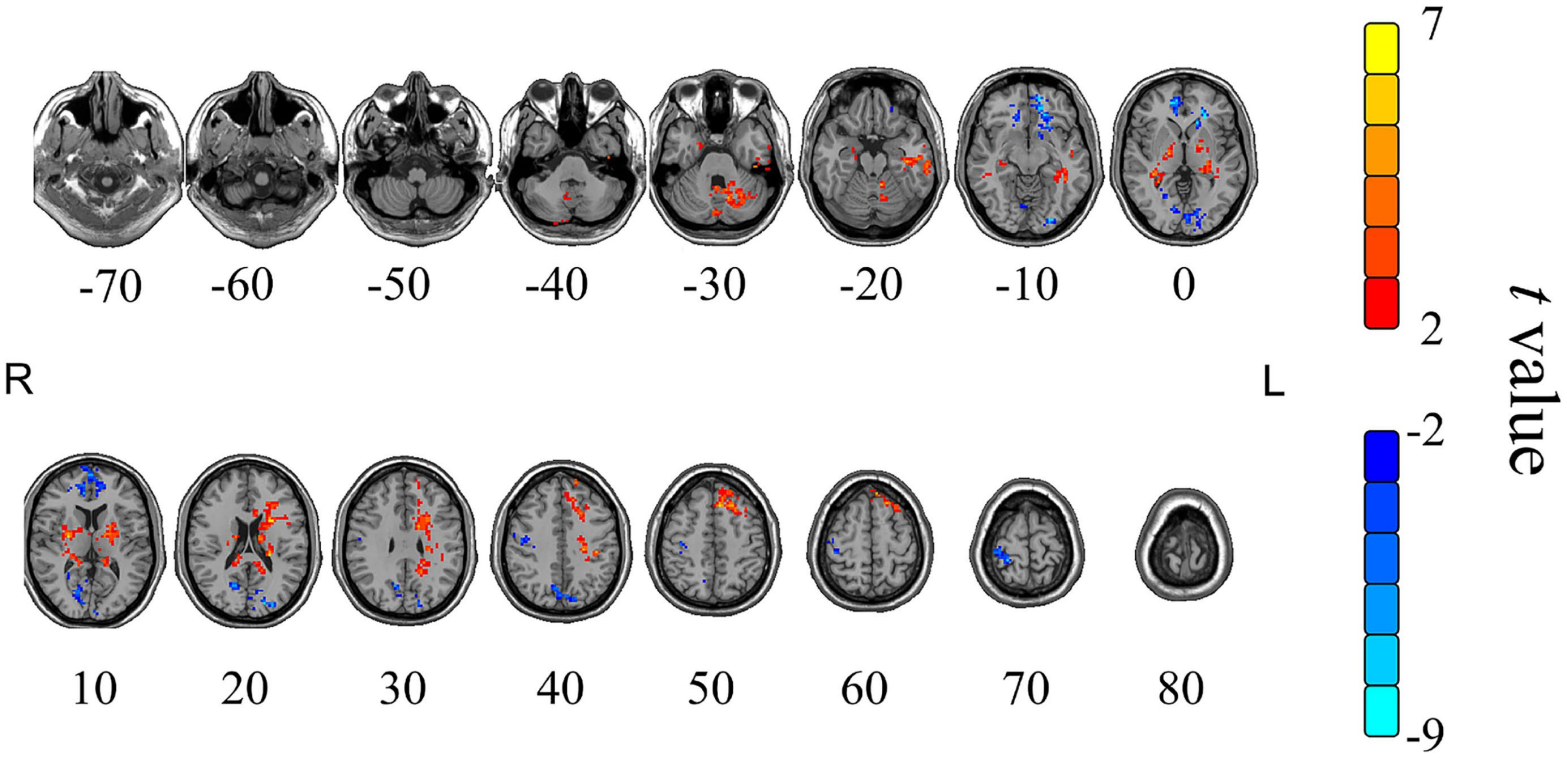
The changes of fALFF value in the HSM group compared to the NHSM group after moxibustion. Color scale denotes the *t*-value; yellow and red represent the brain area with increased fALFF, while blue represents the brain area with decreased fALFF; R represents right in the brain, L represents left in the brain; the paired *t*-tests, single voxel threshold *p* < 0.05, cluster volume threshold for the 85 continuum element K value greater than or equal to 85.

Changes in fALFF after moxibustion between the HSM and NHSM groups are shown in Table 5 and Figure 7. After moxibustion, brain regions with significantly increased fALFF in the heat-sensitive acupoint group compared to the non-heat-sensitive group included the external nucleus, white matter, right hemisphere, left cerebellum, and left hemisphere. Regions with significantly decreased fALFF included the frontal lobe, occipital lobe, and precentral gyrus.

**Table 5:** Cerebral regions with significant changes of fALFF value in the HSM group compared to the NHSM group after moxibustion.

Cerebral regions	Voxels	Peak MNI coordinates			*t*-value (peak)	*P*-value
		*X*	*Y*	*Z*		
Extra nuclear	188	24	−36	15	5.80	<0.001
White substance region	85	−15	−39	12	3.63	0.006
Right cerebrum	196	24	−36	15	5.80	<0.001
Left cerebellum	147	−12	−66	−24	5.30	<0.001
Left cerebrum	264	−60	−3	−27	4.34	0.002
Frontal lobe	275	−24	21	−6	−4.06	0.002
Occipital lobe	311	18	72	6	−4.84	<0.001
Precentral gyrus	112	39	−21	48	−4.43	0.003

MNI coordinates, Montreal Neurological Institute coordinates of the peak voxel; *t*-values: statistical value of the peak voxel, *P* < 0.05; Alphasim correction.

### 3.4 Correlation analysis

**Figure 8:**
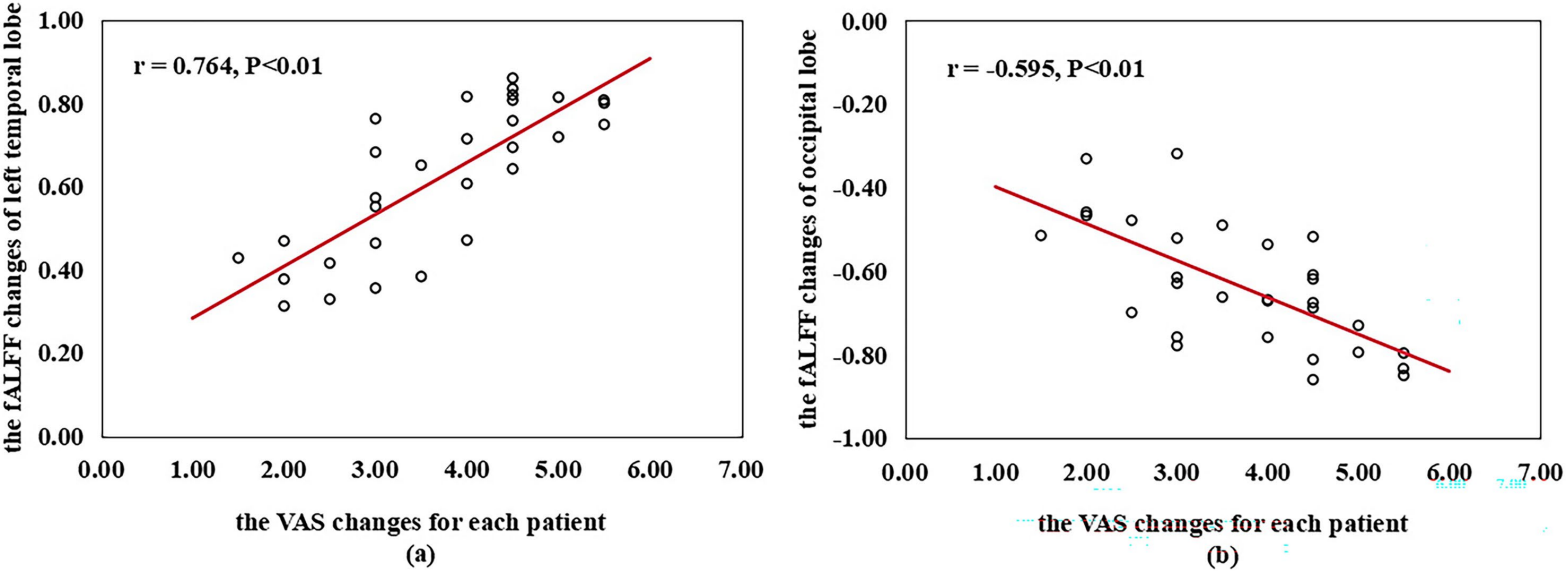
Correlation between the fALFF changes of different ROI regions and the VAS changes for each patient (pre- vs. post-treatment) within the HSM group. **(a)** The VAS changes for each patient (pre- vs. post-treatment) in the HSM group was positively correlated with the fALFF changes of left temporal lobe (*r* = 0.764, *p* < 0.01) and **(b)** negatively correlated with the fALFF changes of the occipital lobe (*r* = −0.595, *p* < 0.01). fALFF, The fractional amplitude of low-frequency fluctuation; ROI, regions of interest; VAS, visual analog scale. Pearson correlation analysis was used with a statistical significance threshold of *p* < 0.05.

As shown in Figure 8a, the fALFF changes of the left temporal lobe were positively correlated with the VAS changes for each patient (pre- vs. post-treatment) in the HSM group (*r* = 0.764, *p* < 0.01). However, as shown in Figure 8b, the fALFF values changes of the occipital lobe were negatively correlated with the changes in VAS scores (pre- vs. post-treatment) in the HSM group (*r* = −0.595, *p* < 0.01). No significant correlation was observed between the fALFF changes in abnormally active brain regions and the VAS changes in NHSM group.

## 4 Discussion

Traditional Chinese Medicine (TCM) interventions include herbal medicine (internal/external), acupuncture, and tuina, among which heat-sensitive moxibustion has gained clinical prominence for its rapid onset, swift analgesia, and sustained efficacy (40). The ST35 (Dubi) acupoint is empirically recognized for resolving swelling, alleviating pain, and unblocking meridians. As a standard protocol for KOA, moxibustion at ST35 enhances thermal penetration and sensitization response, optimizing therapeutic outcomes (41).

The VAS is a reliable tool for assessing pain intensity and can intuitively reflect the analgesic effects of treatment (42). In this study, both the HSM and NHSM groups showed reduced VAS scores after heat-sensitive moxibustion, while the VAS scores reduction in the HSM group (50.4% reduction) was greater than that in the NHSM group (10.5% reduction). This indicates that heat-sensitive moxibustion achieves better pain relief in KOA patients with moxibustion sensation compared to without moxibustion sensation. Our results align with previous clinical research (19): a multicenter randomized controlled trial on knee osteoarthritis similarly found that targeting heat-sensitive points with moxibustion produced significantly greater improvements in both pain and physical function compared to routine moxibustion at conventional, non-sensitive locations. Patients experiencing moxibustion sensation during moxibustion perceive not only superficial thermal stimulation but also a penetrating warmth in deeper muscles or joints, whereas those without moxibustion sensation only experience local warmth. This difference may be attributed to the central nervous system’s reward response to thermal stimulation, which could enhance the analgesic effect (43).

In this study, significant changes of fALFF values were observed in brain regions such as the frontal, temporal, and occipital lobes in both the HSM and NHSM groups. Specifically, the frontal and temporal lobes were significantly activated in the HSM group, while they were significantly inhibited in the NHSM group. In chronic pain states, the brain undergoes neuroplastic changes. The frontal lobe, with its strong cognitive and emotional regulatory functions, can reduce pain unpleasantness via cognitive reappraisal rather than mere sensory-discriminative processing, playing a critical role in chronic pain development (44, 45). Enhanced neural activity in the temporal lobe is associated with stress recognition and memory in chronic pain, primarily responsible for pain cognition and emotional responses (46–49). The significant activation of the frontal and temporal lobes in the HSM group suggests that heat-sensitive moxibustion effectively increases excitability and spontaneous neuronal activity in these regions, thereby modulating pain perception. This pattern of increased activity in cognitive-emotional processing regions aligns with findings from other neuroimaging studies on moxibustion analgesia (50). In contrast, the significant inhibition of these regions in the NHSM group may be related to the need for sustained attention to moxibustion feeling, leading to neural fatigue and reduced excitability (51). The occipital lobe, a higher-order visual processing center, is connected to key regions of the endogenous pain modulation system and participates in regulating spinal dorsal horn neuronal activity (52). Its neural activity is associated with higher-order integration of sensory and emotional functions (53). In this study, both groups showed inhibition in the occipital lobe, indicating that heat-sensitive moxibustion modulates visual input signals and neuronal activity to reduce pain perception in KOA patients. Overall, the HSM group exhibited both increased and decreased spontaneous neuronal activity in brain regions such as the frontal, temporal, and occipital lobes, while the NHSM group primarily showed decreased activity. This differential neural response pattern reflects the modulatory effects of heat-sensitive moxibustion on the pain-default network in KOA patients. Interestingly, the aforementioned moxibustion study on mild cognitive impairment patients also found a bidirectional regulation (both increases and decreases) of ALFF values across multiple brain networks, including the default mode and visual networks, following intervention (54). Specifically, moxibustion at the Dubi (ST35) acupoint appears to achieve its analgesic effects by selectively regulating neural activity in key cognitive-emotional processing regions including the frontal lobe, temporal lobe, and occipital lobe, with the heat-sensitive state enabling more comprehensive neuromodulation that correlates with superior clinical outcomes.

In this study, we found patients with enhanced fALFF in the left temporal lobe demonstrated more significant changes in VAS scores (Figure 8a), suggesting that the mechanism of heat-sensitive moxibustion analgesia may be related to the temporal lobe’s ability to integrate pain. Enhanced temporal lobe activity reflects chronic pain-related stress encoding and memory consolidation (46), facilitating cognitive-affective pain processing (48, 49, 55). This finding aligns with a recent neuroimaging study on moxibustion for mild cognitive impairment (54). Collectively, these findings suggest that heat-sensitive moxibustion enhances neuronal excitability and spontaneous activity across these regions, supporting multidimensional modulation of pain perception. Paradoxically, reduced fALFF in the occipital lobe (a region primarily dedicated to visual and higher-order sensory processing) was also associated with clinical improvement (56). This negative correlation suggests that decreased neural activity in sensory-related cortices may contribute to pain relief, possibly by attenuating the integration or amplification of nociceptive signals within the brain’s sensory processing streams. The association between specific brain region fALFF changes and clinical outcomes is not unique to moxibustion. A 2024 study on acupuncture for chronic neuropathic pain found that increased fALFF in the right superior parietal lobule was not only associated with better pain relief but could also serve as a potential predictor of treatment response (57). This was also corroborated by our correlation analysis, which found that the fALFF changes of the occipital lobe were negatively correlated with the VAS score differences (Figure 8b).

Heat-sensitive moxibustion is widely used in KOA treatment due to its therapeutic benefits (19). Compared to the NHSM group, this study found that the HSM group showed significant activation in brain regions such as the external nucleus, white matter, right hemisphere, left cerebellum, and left hemisphere, while regions such as the prefrontal lobe, occipital lobe, and precentral gyrus were significantly inhibited. The external nucleus is a key brain region involved in pain modulation and participates in the descending pain inhibitory pathway (58). The pain-relieving effects of heat-sensitive moxibustion in patients with KOA may be associated with the activation of this region. White matter participates in pain signal transmission and modulation, influencing pain perception and emotional responses (59). In the HSM group, the white matter area in patients with KOA was activated after heat-sensitive moxibustion, which improves the pain tolerance of patients (60). The cerebrum is a high-level nerve center related to sensation and movement (61), and the cerebellum can sense incoming information and control fine movements (62). Heat-sensitive moxibustion can improve the symptoms of pain, morning stiffness, and unfavorable joint flexion and extension in patients with KOA, which may be related to the activation of nerve activity in the cerebrum and cerebellum. The prefrontal lobe, occipital lobe, and precentral gyrus are involved in pain perception and emotional response, which are regulated by neurotransmitters and modulators, as well as the synergistic effect of brain default mode network (63–67). In the NHSM group, heat-sensitive moxibustion for KOA patients failed to effectively regulate the function of these brain areas, resulting in reduced activity of brain areas related to pain perception and emotional response. In summary, these findings suggest that the differences in the pain-related default network state (activation or inhibition) in patients with KOA are associated with the sensitization of acupoints. Moxibustion during the acupoint sensitization state leads to more significant and extensive activation of brain regions in KOA patients. The analgesic effects of heat-sensitive moxibustion are closely linked to neural regulation in the brain.

## 5 Limitations

Our study has several limitations: (1) The phenotype-driven allocation based on subjective heat sensitivity, while central to our research question, limits the generalizability of our findings and introduces potential expectation bias. Future complementary designs—such as randomizing within phenotypes and employing objective biomarkers like infrared thermography—are needed to validate and extend these findings. (2) The lack of systematic collection of participants’ prior moxibustion history may influence subjective outcomes through expectation effects. Future studies should prospectively incorporate “prior moxibustion history” as an important covariate in design and statistical analysis to better distinguish between the specific and non-specific effects of treatment. (3) while fALFF effectively reflects the intensity of spontaneous neural activity at rest, a more comprehensive and accurate investigation of the central neural mechanisms of heat-sensitive moxibustion analgesia would require combining it with other analytical methods—such as regional homogeneity (ReHo) and functional connectivity (FC) analysis—in future studies to improve the interpretability and reliability of the findings.

## 6 Conclusion

In general, by fALFF analyzing the regional brain functions of patients with KOA using rs-fMRI, we found that the sensitization of acupoints was related to the default mode network state of pain in patients with KOA during the heat-sensitive moxibustion at ST35 acupoint. The frontal, temporal, and occipital lobes demonstrate differential engagement in pain signal processing, with heat-sensitive states eliciting more pronounced and extensive neural activation patterns compared to non-heat-sensitive conditions.

## Data Availability

The original contributions presented in the study are included in the article/supplementary material, further inquiries can be directed to the corresponding author.

## Footnotes

1
www.restfmri.net
